# Bayesian Tomography for Projections with an Arbitrary Transmission Function with an Application in Electron Microscopy

**DOI:** 10.6028/jres.111.031

**Published:** 2006-12-01

**Authors:** Zachary H. Levine, Anthony J. Kearsley, John G. Hagedorn

**Affiliations:** National Institute of Standards and Technology, Gaithersburg, Maryland 20899

**Keywords:** Bayesian tomography, Beer’s Law, maximum likelihood, multiple scattering, photonic band gap, physical transmission function, transmission electron microscope, transmission-thickness relation

## Abstract

The vast majority of the developments in tomography assume that the transmission of the probe through the sample follows Beer’s Law, i.e., the rule of exponential attenuation. However, for transmission electron microscopy of samples a few times their mean free path, Beer’s Law is no longer an accurate description of the transmission of the probe as a function of the sample thickness. Recent simulations [Z. H. Levine, Appl. Phys. Lett. **82**, 3943 (2003)] have demonstrated accounting for the correct transmission function leads to superior tomographic reconstructions for a photonic band gap sample 8 µm square.

Those recent simulations assumed that data was available at all angles, i.e., over 180°. Here, we consider a limited-angle case by generalizing the Bayesian formalism of Bouman and Sauer to allow an arbitrary transmission function. The new formalism is identical to that of Bouman and Sauer when the transmission function obeys Beer’s Law. The examples, based on 140° of data, suggest that using the physical transmission function is a requirement for performing limited angle reconstructions.

## 1. Motivation and Approach

Tomography became an important field about 40 years ago with the application x-rays to human subjects to detect tumors [1]. In the classical Computerized Axial Tomography (CAT) scan, a line of x-ray sources and detectors are rotated about the patient. The practice quickly spread to electron microscopy, although in this case the sample rather than the beam is rotated. The principal contrast mechanism in x-ray scattering is absorption which follows Beer’s Law, i.e., the rule of exponential attenuation. Although it was necessary to develop radically different algorithms for tomography using magnetic resonance imaging or ultrasound [2], the electron microscopy community imported the assumptions of a probe traveling in a straight line through a sample with exponential attenuation. The assumption is valid for thin samples [3]. It was long recognized that for thick samples multiple scattering would play a role and render these assumptions invalid [4]. Recently, Levine noted that near the onset of multiple scattering (as the sample thickness under consideration increases), there is a regime in which the projective assumption remained valid, but the transmission as a function of thickness deviated significantly from Beer’s Law [5,6]. In particular, in simulation excellent reconstructions of an 8 µm square sample of a photonic band gap material were achieved using the multiple scattering transmission function [5]. The work assumed that scans were available through a tilt angle of 180°, and the filtered backprojection algorithm was used.

In practice, it is often not possible to obtain data throughout a 180° angular range; in practice, data are acquired over a range of 90° to 160°. In this case, the filtered backprojection algorithm is subject to strong streak artifacts. Such an algorithm amounts to zeroing out the Fourier components of the reconstructed image in the unmeasured angular range. The issue of how to recreate the missing information has been a lively topic for at least two decades. One approach is the constraint-based projection onto convex sets (POCS) [7]. Various forms of regularization have been considered [1]. Here, we will follow the lead of Bouman and Sauer and use the Bayesian approach known as the generalized Gaussian Markov random field (GGMRF) [8]. The principal features of the GGMRF formalism are: a prior distribution based on correlations of neighboring pixels (or voxels in 3D) in which the smoothness of the reconstruction may be adapted to the sample and a quadratic approximation to the log of the likelihood derived from Poisson statistics. In their formulation, Sauer and Bouman assume the Beer’s Law relation [9]. Here, we will make a more general assumption: that the transmission is any known function, with sufficient differentiability, of a linear combination of the material parameters of the sample. A projection is a special case of this general relation which happens to lower the algorithmic complexity. The transmission functions derived from multiple scattering theory [5,6] represent the special case which motivated the development of the present formalism.

We will generalize the formulation of Bouman and Sauer [8,9]. We will reconstruct the density of the material in each pixel; these real parameters are collectively called *f*, or *f_i_* for an individual component. Various observations are made—in practice, these are projections often indexed by a tilt angle *θ_j_* and an offset parameter *τ_j_*, but other parameterizations could be indexed within the theory. (The (*θ_j_*, *τ_j_*) will be distinct only when considered pairwise.) Collectively the observations are denoted by *n*, and individually by *n_j_*. The solution of the maximum likelihood (ML) problem is

$${\hat{f}}_{ML}=\arg\underset{f}{\max}L_{0}(n|f)$$ (1)

where *L*_0_ is the logarithm of the likelihood. Constructing *L*_0_ requires both a physical model to relate the material parameters *f* to the observations *n* and statistical assumptions; here, the observations are taken to be counts with a Poisson distribution. The presence of the monotonic function ln does not shift the maximum argument compared to the likelihood exp *L*_0_(*n*|*f*). In the Bayesian case, specifically maximum *a posteriori* (MAP) estimation, we wish to maximize the joint probability over the possible reconstructions *f* with the observations *n* held fixed, i.e.,

$${\hat{f}}_{MAP}=\arg\underset{f}{\max}L(n|f)$$ (2)

where *L*(*n*, *f*) is the log-likelihood of the joint *a priori* probability distribution. The joint log-likelihood may be expressed as a sum of the conditional log-likelihood of (1) and the prior probability distribution *g*(*f*), hence

$${\hat{f}}_{MAP}=\arg\underset{f}{\max}\{L_{0}(n|f)+\ln\;g(f)\}.$$ (3)

If *g*(*f*) is assumed to be a constant, then the MAP reduces to the ML. In the present work, we retain the GGMRF prior distribution of Bouman and Sauer [8], i.e.,

$$\log\;g(f)=-\lambda^{p}\left(\sum_{i}a_{i}|f_{i}{|}^{p}+\sum_{\langle ik\rangle }b_{i,k}|f_{i}-f_{k}{|}^{p}\right);$$ (4)

here, 〈*ik*〉 defines a sum over pairs of pixels in the various neighborhoods, 1 < *p* ≤ 2, and λ, *a_i_*, and *b_i,k_* are positive definite constants. The property *b_i,k_* = *b_k,i_* is required to obtain a Markov random field.

Only the ML term [9] will be modified. Sauer and Bouman [9] proposed a quadratic approximation to the log-likelihood *L* and show that it is an excellent approximation for the range of counts typically employed in tomography. Following Sauer and Bouman, we expand the log-likelihood to second order as

$$L(n|f)\approx L(n|f^{\left(0\right)})+\sum_{k}\frac{\partial L(n|f^{\left(0\right)})}{\partial f_{k}}(f_{k}-f_{k}^{\left(0\right)})+\frac{1}{2}\sum_{ik}\frac{\partial^{2}L(n|f)}{\partial f_{k}\partial f_{i}}(f_{k}-f_{k}^{\left(0\right)})(f_{i}-f_{i}^{\left(0\right)}).$$ (5)

For a function with a minimum, we may choose *f*^(0)^ such that

$$\frac{\partial L(n|f^{\left(0\right)})}{\partial f_{k}}=0.$$ (6)

So *f* ^(0)^ = *f_ML_*. The log-likelihood *L* is taken to be that associated with the Poisson distribution

$$L(n|f)=\sum_{j}\ln n_{j}!-n_{j}\ln[I_{j}{\bar{T}}_{j}(f)]+I_{j}{\bar{T}}_{j}(f)$$ (7)

where we introduce the dosage *I_j_*, defined as the Poisson parameter governing the number of particles entering the sample for scan parameters *j* and the transmission function for each observation ${\bar{T}}_{j}(f)$. When the log-likelihood has the form of (7), the gradient components of the log-likelihood may be expressed as

$$\frac{\partial L(n|f^{\left(0\right)})}{\partial f_{k}}=\sum_{j}\left(I_{j}-\frac{n_{j}}{{\bar{T}}_{j}(f)}\right)\frac{\partial {\bar{T}}_{j}(f)}{\partial f_{k}}.$$ (8)

We elect to expand about a solution to (6) which obeys

$$n_{j}=I_{j}{\bar{T}}_{j}(f^{\left(0\right)})$$ (9)

although other solutions may exist in particular cases.

The Hessian of the log-likelihood is given by

$$\frac{\partial^{2}L(n|f)}{\partial f_{k}\partial f_{i}}=\sum_{j}\left[\frac{n_{j}}{{\left[{\bar{T}}_{j}(f)\right]}^{2}}\frac{\partial {\bar{T}}_{j}(f)}{\partial f_{k}}\frac{\partial {\bar{T}}_{j}(f)}{\partial f_{i}}+\left(I_{j}-\frac{n_{j}}{{\bar{T}}_{j}(f)}\right)\frac{\partial^{2}{\bar{T}}_{j}(f)}{\partial f_{k}\partial f_{i}}\right].$$ (10)

At *f* = *f*^(0)^, the final term vanishes due to (9), so

$$\frac{\partial^{2}L(n|f^{\left(0\right)}}{\partial f_{k}\partial f_{i}}=\sum_{j}\frac{n_{j}}{{\left[T_{j}(f^{\left(0\right)})\right]}^{2}}\frac{\partial \bar{T}(f^{\left(0\right)})}{\partial f_{k}}\frac{\partial \bar{T}(f^{\left(0\right)})}{\partial f_{i}}.$$ (11)

When we perform reconstructions from projections, it is useful to call *P_j_* the projection, defined to be the sums over the reconstructed pixels

$$P_{j}=\sum_{i}A_{ji}f_{i}$$ (12)

where *A* is a matrix which defines the projections. In the examples in this work, we follow the common practice [1] of taking *A_ji_* to be the length a given line indexed by *j* is inside a given pixel indexed by *i*. Although we do not require it for the general theory, in practice *A* will be a sparse matrix. Nor do we need to assume anything about the projections representing approximations to straight line integrals, although that key case is certainly included. We assume that

$${\bar{T}}_{j}(f)=T_{j}(P_{j})$$ (13)

where *T_j_* is a scalar function of a scalar variable; *T_j_* will be known for a particular reconstruction problem. The components of the gradient of (13) are given by

$$\frac{\partial T_{j}(P_{j})}{\partial f_{i}}=T_{j}^{\prime }(P_{j})A_{ji}$$ (14)

where the prime denotes the derivative with respect to the argument. When (12) and (13) apply, (14) allows us to rewrite (11) as

$$L(n|f)\approx L(n|f^{\left(0\right)})+\frac{1}{2}\sum_{j}n_{j}\times {\left(\frac{d\ln T_{j}(P_{j}^{\left(0\right)})}{dP_{j}}\right)}^{2}{\left(\sum_{i}A_{ji}f_{i}-P_{j}^{\left(0\right)}\right)}^{2}$$ (15)

where $P_{j}^{\left(0\right)}=\sum_{i}A_{ji}f_{i}^{\left(0\right)}$ in analogy with (12). From (9) and (13), we find $P_{j}^{\left(0\right)}=T_{j}^{-1}(n_{j}/I_{j})$; note that we have knowledge of $P_{j}^{\left(0\right)}$ for all *j* without knowledge of the values $f_{i}^{\left(0\right)}$ individually.

Beer’s Law may be introduced at this stage by making the association *T_j_*(*z*) = *e*^−^*^z^*. In this case,

$$\frac{d\ln T_{j}(z)}{dz}=-1$$ (16)

and (15) reduces to Eq. (9) of Sauer and Bouman [9]. Since the prior is unchanged, (3) will reduce to the GGMRF of Bouman and Sauer [8] as well. Because we retain the quadratic approximation of Sauer and Bouman [9], the convergence proofs of Bouman and Sauer [8] should apply to the generalized formalism presented here as well.

In the example of the present work the transmission functions are independent of the observation index *j*, i.e., *T* = *T_j_*. However, we retain *T_j_* so that the final formula will be applicable to the case of heterogeneous detectors. For example, many electron microscopes record a bright-field signal and a dark-field signal simultaneously (*j* would then represent the scan parameters and the detector channel); this work will allow both data sets to be exploited once a calibration of the transmitted signal as a function of the material thickness is performed, say on a well-characterized wedge sample, assuming the correlations between the two channels may be neglected. Note that we did not assume *T_j_*(*P_j_*) was a monotonic. However, when the slope vanishes, the observations have zero weight in the likelihood. Dark-field detectors will have a single peak for the case of scattering through amorphous samples.

## 2. Algorithm

An implementation of the limited-memory Broyden, Fletcher, Goldfarb, and Shanno (L-BFGS) method was employed to solve our large-scale unconstrained optimization problem [10]. The L-BFGS method is often viewed as an extension of the conjugate gradient method where a small amount of additional storage can accelerate the convergence. The L-BFGS method can also be seen as storage-restricted version of the BFGS quasi-Newton method [11]. The method is particularly attractive for the problem at hand because the cost of each iteration of the algorithm and the storage used can be controlled by the user. First derivatives must be computed to evaluate the gradient, but no *a priori* knowledge of the second derivatives is required, although an approximate Hessian matrix is determined as the algorithm proceeds.

In our implementation, the pixels are arranged in a simple square lattice. We find the pixels associated with each observation using the algorithm of Amanatides and Woo [12]. The neighborhood of each pixel is taken to include all the pixels with a common edge (but not merely a common corner). We augment the pixels whose material densities are to be determined by a surface of pixels which are fixed to have density zero. We take the *a_j_* in (4) to be 0, as suggested by Bouman and Sauer [8], and the single scalar value *w* is given by w = –λ*^p^b_j,k_*, independent of *j* and *k*.

If we consider a domain of size *N^d^*, where *N* is the number of pixels along an edge of the domain and *d* is the dimension of physical space, assuming that *A_ji_* is non-zero for *O*(*N*) values of *i* (i.e., pixels) for each observation *j*, it is possible to compute the gradient of the log-likelihood in *O*(*N^d^*^+1^) steps and the prior in *O*(*N^d^*) steps. The situation is similar for local updates: each of *N ^d^* pixels typically is influenced by *O*(*N*) observations, so updating the log-likelihood will take *O*(*N^d^*^+1^) steps for a full sweep through the grid. Assuming the local update scheme is augmented by a multigrid method, we expect the two classes of algorithms to yield comparable run times. However, we favor the global updates because of the relative simplicity of the coding and the possibility of a straightforward extension to cases in which the geometry or observations are heterogeneous. Note also that the iterations of (15) require no more time or storage than the original method of Bouman and Sauer [8,9] because the $P_{j}^{\left(0\right)}$ are known.

## 3. A Representative Example

**Fig. 1:**
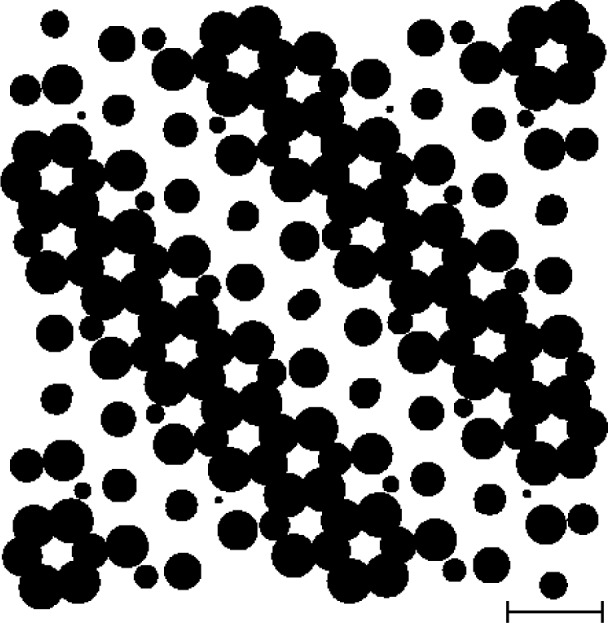
The simulated target (or “phantom”) is a finite section of a quasiperiodic pattern of columns of a polymer which arise from cutting a 3D model of a photonic band gap crystal at an irrational angle and extending the exposed plane translationally in the third dimension. The scale bar is 1 µm. The material is dark. The void is light. The figure is a subset of the one used in Ref. 5.

As an example, we consider a simulation based on a photonic band gap crystal. A 6 µm by 6 µm material and void pattern is shown in Fig. 1; this is our test object or “phantom.” The image has various features which make it a non-trivial test case: small and large circles, which may be slightly overlapping or slightly split, in addition to having a global inhomogeneity in the form of approximate stripes running parallel to the *x* = –*y* line in the sample. Moreover, the system is several times larger than those used typically in electron microscopy. The developments in this paper are motivated by enabling experiments in this regime.

**Fig. 2:**
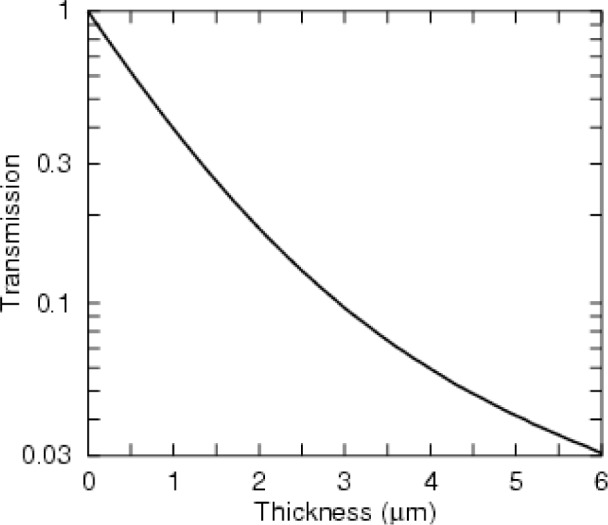
The transmission function was derived from a Monte Carlo simulation of the 4 million 300 keV electrons passing through a polymer into a bright-field detector with a 10 mrad half-angle of acceptance. Multiple scattering manifests itself from the significant deviations compared to Beer’s Law, i.e., a straight line on this semi-log plot. See Ref. 5 for further details.

The response of a typical bright-field detector of 300 keV electrons through a polymer material [5] is given in Fig. 2. The key feature of the transmission curve is that the detected signal deviates considerably from Beer’s Law. A considerable amount is understood about the relationship of the transmission to the underlying cross sections and the functional form of the transmission curve [6], but for the present, all that is required is the transmission function and its logarithmic derivative.

Scan data was obtained through Monte Carlo multiple scattering, using a code presented earlier [5,6]. The data was collected at 192 angles uniformly distributed over 140°. There are 361 uniformly spaced offset parameters (τ values) at each tilt angle, and a 1000 electrons per scan parameter (a tilt-angle-offset pair) were simulated.

We performed a MAP reconstruction using (3), and a maximum likelihood reconstruction using (1). We also found a reconstruction using the unmodified Bouman-Sauer formalism, i.e., using Beer’s Law instead of the transmission function given in Fig. 2. The result bears no relationship to the phantom [13]. However, in other test cases, described elsewhere, [13] in which scan data was generated using Beer’s Law, the code performed very well, so we attribute the poor reconstruction to the use of the wrong transmission function. The poor performance of Beer’s Law is in contrast to the case in which the filtered backprojection algorithm was used on the same sample with 180° of data and gave a reconstruction with global artifacts but captured most of the medium and fine structure of the phantom [5].

**Fig. 3:**
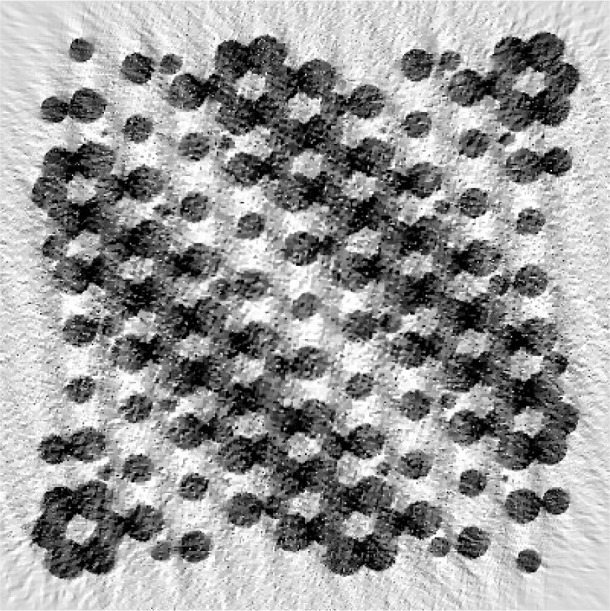
Reconstructed reverse gray scale image of Fig. 1 using the Bayesian approach outlined in the text. The image has 400×400 pixels; observations were taken with 361 uniformly spaced offset values at 298 angles distributed uniformly over a 140° range; 226 iterations were used. The parameters *p* = 1.1 and *w* = 0.002 were selected. The saturation values are taken to be the values at the 5th and 95th percentiles of the pixels in the image.

The Bayesian reconstruction is shown in Fig. 3. The reconstruction was performed using the parameters *p* = 1.1, which is known to give good results for the material reconstruction problem [8] and *w* = 0.002. The value for *w* was chosen from the set {0.001, 0.005, 0.01, 0.1, 0.2, 0.4} by a subjective optimization of the quality of the reconstructed image. The ML reconstruction is equivalent to *w* = 0. The choices *w* = 0.001, 0.005 or 0.01 yield reconstructions very similar to the one presented; *w* = 0.1 and 0.2 lead to a considerable fusion of the fine structures, but correctly represent all the large circles. The case *w* = 0.4 fails to give a recognizable reconstruction. Convergence to near machine precision (10^−12^) was achieved in 226 iterations using the L-BFGS algorithm, restarting the construction of the approximate Hessian matrix after every sixth iteration. No attempt was made to optimize the convergence. It took approximately 13 minutes to perform this reconstruction on a 2.8 GHz computer.

The reconstruction is generally of a very acceptable quality. In particular, the reader may wish to examine the presence of some of the smaller circles in the original target presented in Fig. 1 in the reconstruction of Fig. 3 as well as some of the narrow separations. Certain artifacts are also seen to be present, such as the vertical whitish stripes indicating a lack of density.

**Fig. 4:**
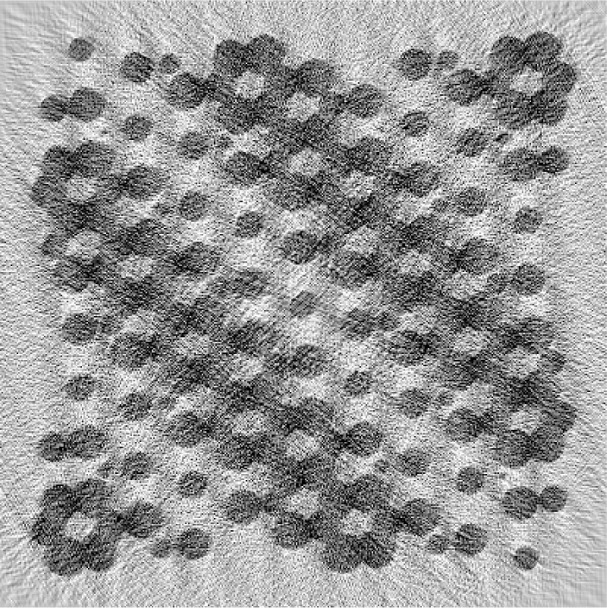
Reconstruction similar to Fig. 3, using 224 iterations of the maximum likelihood objective function.

**Fig. 5:**
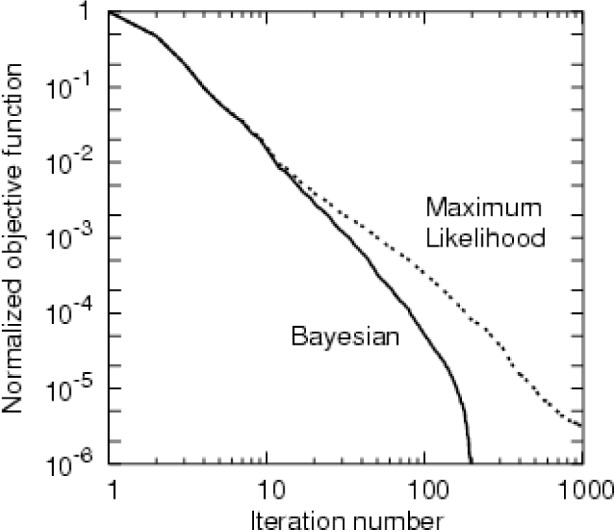
The objective function is normalized by mapping the range of the fully converged value to the initial value to 0 to 1. The fully converged value is taken from iteration number 226 for the Bayesian case (when very stringent convergence criteria were satisfied) and iteration number 4200 for the maximum likelihood case.

**Fig. 6:**
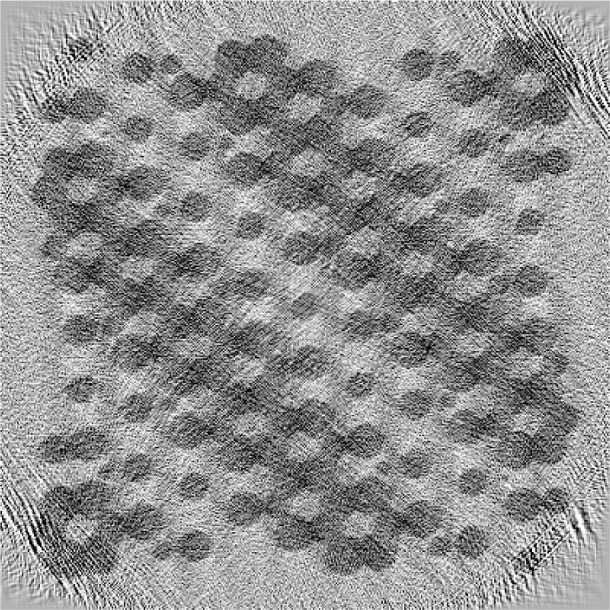
Reconstruction similar to Fig. 3, using 4200 iterations of the maximum likelihood objective function.

The ML result with 224 iterations, approximately the same number of iterations as used for Fig. 3, is shown in Fig. 4. Less convergence was achieved in this case, as illustrated in Fig. 5. The reconstruction of Fig. 4 gives the features of the phantom nearly as well as Fig. 3. However, the contrast is lower. The ML case was allowed to run for 4200 iterations; the result is presented in Fig. 6. Curiously, the result is worse, although the objective function is lower. The artifacts build gradually through the course of the iterations. We attribute the behavior to the property of the L-BFGS algorithm that it resolves lower spatial frequencies before higher ones. So the partially converged result represents a kind of low-pass filter of the more fully converged result, and hence is less noisy. The Bayesian prior itself is a regularization or low-pass filter which explains the rapid convergence of the L-BFGS procedure in this case. Our findings are similar to those of Sauer and Bouman [9] who found a local-update algorithm (which they denote “Gauss-Seidel”) outperformed conjugate gradient (which, like L-BFGS, is a global-update algorithm) for the maximum likelihood case, but was comparable for the MAP case. The local-update algorithm converges the high-frequencies best and may need to be augmented by multigrid methods [14] to achieve good low-frequency behavior.

## 4. Summary

We have presented a generalization of the quadratic approximation to the log-likelihood of Sauer and Bouman [9] which may be applied to any transmission function, not only Beer’s Law as assumed in the original work. The new formulation reduced to the old in the case in which the transmission function is Beer’s Law. The new formalism may be applied to the generalized Gaussian random Markov field model of Bouman and Sauer [8]. We have demonstrated through Monte Carlo simulation that the new formalism may be applied to a limited-angle tomographic tilt series acquired from a scanning transmission electron microscope on a sample, which is large compared to the usual practice in electron tomography. The GGMRF yields a superior image to a maximum likelihood reconstruction in the case studied.
